# Drone Infrared Thermography for Detecting Skin Thermal Anomalies in Bottlenose Dolphins: Preliminary Insights

**DOI:** 10.1002/ece3.72892

**Published:** 2026-01-26

**Authors:** Charlie White, Andrew P. Colefax, Guido J. Parra

**Affiliations:** ^1^ Cetacean Ecology, Behaviour and Evolution Lab, College of Science and Engineering Flinders University Adelaide South Australia Australia; ^2^ Sci‐Eye Goonellabah New South Wales Australia; ^3^ Queensland Department of Environment, Tourism, Science and Innovation Brisbane Queensland Australia

**Keywords:** bottlenose dolphin, drone‐IRT, infrared thermography, marine mammal health, thermal imaging

## Abstract

Monitoring the health of cetaceans is challenging as traditional approaches including vessel‐based surveys and necropsies are often opportunistic and limited in their ability to detect subtle physiological changes. Infrared thermography (IRT) offers a non‐invasive alternative by detecting surface temperature anomalies that may reflect localised physiological variation, including changes associated with inflammation, scarring, tissue disruption or thermoregulatory processes. Mounted on drones, IRT can enable remote thermal imaging of free‐ranging individuals. This case study presented preliminary observations from the exploratory use of drone‐IRT to detect localised thermal anomalies in the skin of bottlenose dolphins ( *Tursiops truncatus* ) under human care. A total of 14 adult dolphins were monitored across the Austral summer and winter, with two individuals exhibiting consistent thermal hotspots 3°C–5°C warmer than surrounding body surface temperatures. One individual exhibited a transient anomaly that resolved over time, whereas the other displayed persistent hotspots that became more pronounced. These anomalies corresponded with external markings, suggesting localised alterations in skin surface thermal patterns. This case study provided preliminary evidence that drone‐IRT can detect localised thermal anomalies in a dolphin's skin and highlights the potential for drone‐IRT as a non‐invasive tool for monitoring health in both managed and wild dolphin populations. Further quantitative investigations with larger sample sizes and concurrent veterinary assessments may provide validation regarding such observations and to evaluate whether such anomalies are indicative of underlying health issues.

## Graphical abstract

This study presents preliminary observations using drone‐based infrared thermography (drone‐IRT) to detect localised skin temperature anomalies in bottlenose dolphins ( *Tursiops truncatus* ) under human care. Across 14 adults monitored, two individuals exhibited consistent hotspots 3°C–5°C warmer than surrounding body surfaces, corresponding with external markings indicative of irritation or healing. These findings demonstrate the potential of drone‐IRT as a non‐invasive tool for monitoring skin condition and health in cetaceans.

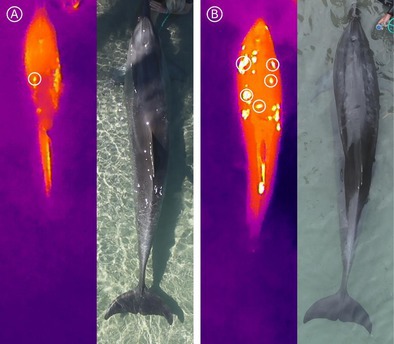

## 1 Introduction

Monitoring the health of wild cetaceans is critical for understanding both individual well‐being and the broader impacts of environmental and anthropogenic stressors. Given their ecological role as upper trophic‐level predators, and being a sentinel species sensitive to environmental change, cetaceans offer important insights into ecosystem health (Bossart 2011). However, real‐time physiological monitoring remains challenging due to their mobility, wide‐ranging patterns and the protected status of many populations, as well as constraints imposed by the aquatic environment. Therefore, assessing cetacean health through non‐invasive approaches can play a vital role in conservation and welfare research, particularly for detecting early signs of trauma, disease or physiological stress.

Traditional methods for assessing health in wild cetaceans have largely relied upon visual surveys from vessels, necropsies or close‐range sampling opportunities (Barratclough et al. 2019; Bossart et al. 2017; de Mello et al. 2025). While valuable, these approaches are often opportunistic, logically constrained and limited in scope or data reliability (Hunt et al. 2013; Van Bressem et al. 2009). In particular, subtle physiological changes, such as early signs of inflammation or infection, are unlikely to be detected through visual observations alone (Cilulko et al. 2013), highlighting the need for supplementary tools that offer greater sensitivity without increasing disturbance to animals.

External signs of injury or trauma, such as scarring, abrasions or swelling, are among the few external indicators of health that can be observed in wild animals. These can result from natural causes (e.g., intra‐species aggression or predation attempts) or anthropogenic interactions (e.g., entanglement or vessel strikes) (Leone et al. 2019; Nicholls et al. 2023; Scott et al. 2005). Such injuries may be accompanied by inflammation or altered tissue physiology, yet current observation methods are limited in their ability to detect or monitor these physiological processes in free‐ranging individuals (Lonati et al. 2022; McCafferty 2007).

Infrared thermography (IRT) is a non‐invasive imaging technique that detects infrared radiation to produce thermal images that reflect surface temperature patterns (Usamentiaga et al. 2014). In recent years, IRT has been used in both medical and veterinary contexts as a tool for assessing health as it can visualise temperature variations that signal underlying physiological processes (Liu et al. 2025; Nääs et al. 2014; Speeckaert et al. 2024). This enables the identification of localised temperature anomalies, which are discrete areas where the surface temperature deviates significantly from the surrounding tissue baseline. In humans, thermal imaging has been used to monitor post‐operative healing and detect infections in surgical wounds (Fridberg et al. 2024). For animals, infrared thermography has been applied across a broad range of veterinary and wildlife contexts to assess health status, injury, vascular change, disease processes and physiological condition, including applications in domestic livestock, wildlife and animals under human care (Dunbar et al. 2009; Hurley‐Sanders et al. 2012; Lin et al. 2018; Mota‐Rojas et al. 2022; Rekant et al. 2016).

The effectiveness of IRT is driven by established physiological responses to inflammation, notably increased metabolic activity and vasodilation, which elevate surface temperatures in affected tissues (Casas‐Alvarado et al. 2024; Ramirez‐GarciaLuna et al. 2022). These thermographic changes can be visualised externally using thermal imaging, enabling real‐time detection of injury or abnormality (Fridberg et al. 2024; Usamentiaga et al. 2014). The ability to visualise such changes in real time, and without physical contact, makes IRT especially valuable for wildlife studies where direct handling may not be feasible.

When integrated with drones, thermal cameras enable remote imaging of wildlife, opening new possibilities for health surveillance in free‐ranging marine mammals. Drones are now widely used in marine mammal research, particularly for morphometric studies and body condition assessments (Bierlich et al. 2021; Christiansen et al. 2016; Christie et al. 2021; Napoli et al. 2024). Therefore, incorporating IRT into these workflows can extend their utility by providing physiological data alongside morphological and behavioural observations. Crucially, drone‐IRT can be applied with minimal disturbance and stress, allowing for repeated assessments across individuals and animal groups.

Despite this promise, the application of drone‐IRT for marine mammal health assessment is still emerging. While foundational studies have demonstrated its potential for diagnosing pathology based on post‐cranial heat anomalies in North Atlantic right whales ( *Eubalaena glacialis* ) (Lonati et al. 2022), this capability has not been systematically evaluated. In this study, we present preliminary observations on the application of drone‐IRT to identify body surface inflammation and scar‐associated elevated heat signatures in bottlenose dolphins ( *Tursiops truncatus* ). Using drone‐IRT footage, we identified two individuals exhibiting distinct, localised thermal anomalies as temporary hotspots coinciding with visible surface body wounds. These observations suggest that drone‐IRT can identify inflammation and tissue injuries near the skin surface in delphinids. While limited by sample size, these findings provide preliminary support for the use of drone‐IRT as a remote health surveillance tool of marine mammals and highlight the potential for further development of this technique in veterinary and conservation contexts.

## 2 Methods

Drone‐IRT data were collected during the 2023 Austral summer (February and March) and winter (July and August) at Sea World, Gold Coast, Australia. Data collection was part of a broader ground‐truth validation study evaluating the accuracy and precision of drone‐IRT (White et al. 2026). The managed setting provided controlled opportunities to capture repeatable imagery across individuals, while still simulating conditions relevant to free‐ranging populations. A total of 14 adult bottlenose dolphins ( *Tursiops truncatus* ) were monitored during the validation study.

Drone flights were conducted using a DJI Matrice 30T (M30T) (DJI 2024), equipped with a gimballed, integrated 3‐in‐1 payload comprising an infrared camera (640 × 512 resolution, 30 Hz), a 12 MP wide‐angle and a 48 MP zoom camera. Infrared and visible (RGB) images were captured simultaneously (within 0.1 s of each other) to allow alignment of thermal anomalies with external markings. Flights were only conducted during daylight hours in winds under 12 m·s^−1^. As part of the validation study, thermograms at different flight altitudes were captured; however, for this study only images from an altitude of 10 m, a camera angle of 0° (zenith) and a setting where the dolphin's dorsal region was fully out of the water were used to provide the most reliable results for temperature measurements (White et al. 2026). All data collection was conducted in the presence of an experienced marine mammal veterinarian and in accordance with animal ethics approval (Flinders University AEC BIOL5505).

Thermal images were processed in FLIR Thermal Studio (version 2.0.6) (Teledyne 2023). Hotspot anomalies were defined as discrete, localised regions of elevated surface temperature that exceeded the adjacent body surface temperature by ≥ 1°C, were visible across consecutive frames, coincided with external skin markings in paired RGB images and were not present across all individuals. This threshold was chosen based on established applications of IRT in veterinary and clinical medicine, where temperature differentials of approximately 1°C are recognised as physiologically significant indicators of inflammation, infection or changes in tissue viability (Fridberg et al. 2024; Lin et al. 2018; Soroko et al. 2013). While footage was not specifically taken to identify these abnormalities, the completion of two different study seasons showed both the recovery and evolution of multiple hotspots on multiple dolphins.

The temperature of each hotspot anomaly was quantified following a standardised process. For images with a single hotspot or a few discrete hotspots, the visibly warmest region was identified and the maximum hotspot temperature was recorded using the spotmeter tool positioned at the centre of the hotspot. To establish a reliable body surface temperature baseline, the adjacent body surface temperature was averaged using a line tool placed along the dorsal surface, extending from the pixel below the blowhole to the pixel above the base of the dorsal fin's leading edge. If a hotspot overlapped this line, the measurement line was ended before the hotspot occurred. Where multiple widespread hotspots were present, the maximum temperatures of the five visibly hottest anomalies were measured and averaged to generate a single representative value for comparison. In all cases, paired comparisons were made between the hotspot and adjacent body measurements within the same image, and summary statistics (mean, range and standard deviation) of the temperature differentials were calculated for each individual. Differences between hotspots and body surface temperatures were assessed using non‐parametric Wilcoxon signed‐rank tests, as Shapiro Wilk tests indicated non‐normality in data residuals (*p* < 0.05). All statistical analyses were performed in R (version 4.3.3) (R Core Team 2024) using base stats functions.

## 3 Results and Discussion

Out of the 14 adult dolphins monitored, all were under routine veterinary care and were considered healthy with no signs of irritation, infection or injury at the time of imaging. Thermal anomalies were detected in two individuals: a 20‐year‐old male (TM5) and a 21‐year‐old female (TF5). TM5 had an average body mass of 236–244 kg across monitoring periods and a total body length of 2.79 m, while TF5 had an average body mass of 222–234 kg and a total body length of 2.70 m. These anomalies were interpreted as localised deviations in skin surface temperature, which may be associated with processes such as acute inflammation or tissue injury, based on abnormal or asymmetrical thermal patterns reflecting changes in peripheral blood flow, localised inflammation or underlying pathology (Cilulko et al. 2013; McCafferty 2007; Rekant et al. 2016). However, in the absence of veterinary ground‐truthing, these patterns cannot be attributed to specific pathological causes or injuries. No comparable persistent or localised thermal anomalies were observed in the remaining 12 dolphins during the study period.

**FIGURE 1:**
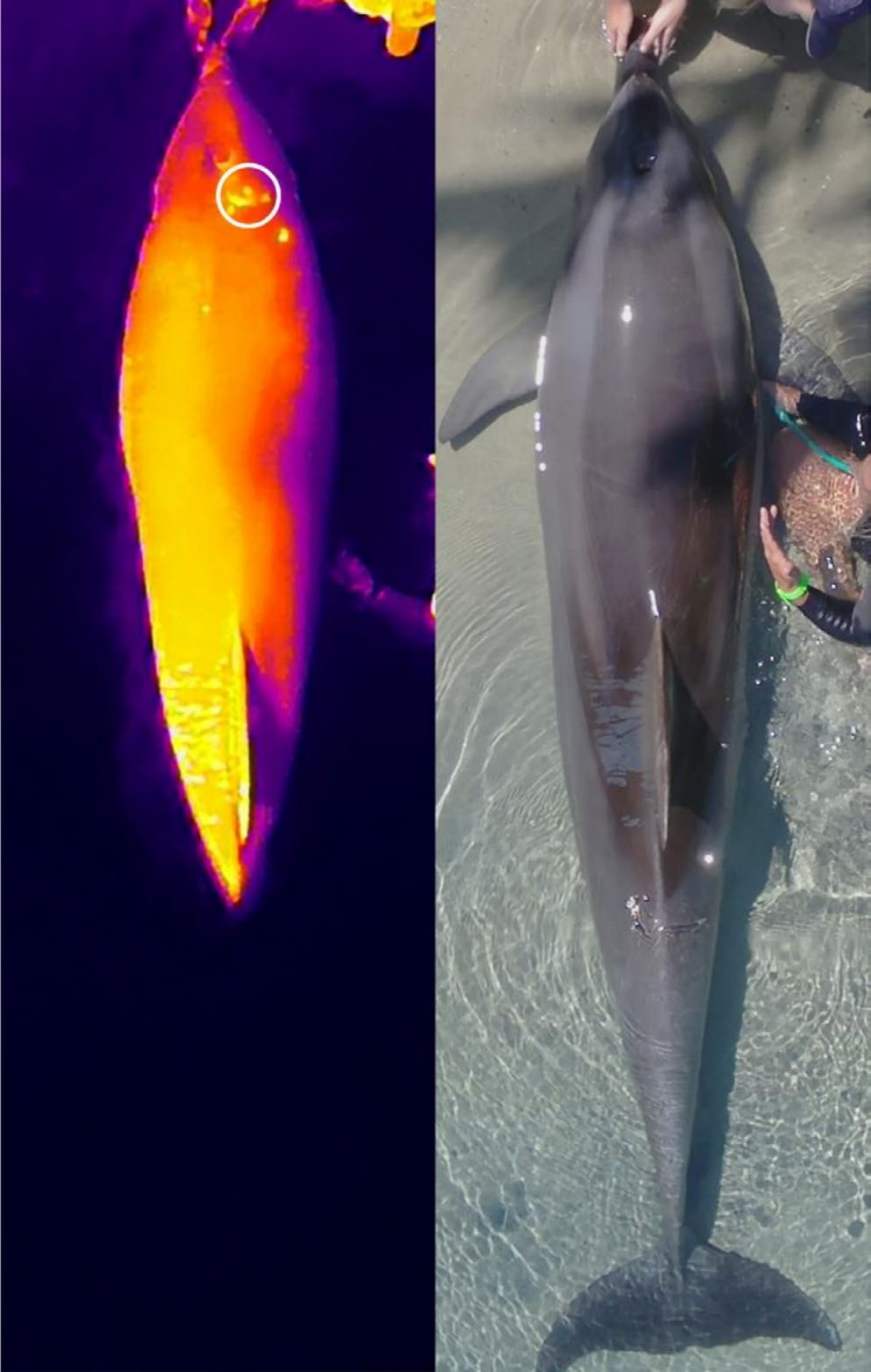
Infrared thermography (left) and paired RGB image (right) of an adult bottlenose dolphin (TM5) recorded during summer 2023 at Sea World, Gold Coast, Australia, captured from a flight height of 5 m and subsequently cropped and zoomed for visual clarity. A distinct crescent‐shaped thermal anomaly is visible posterior to the blowhole in the infrared image, corresponding to the region where scratch marks subsequently developed. Anomaly measured is shown in the white circle.

**FIGURE 2:**
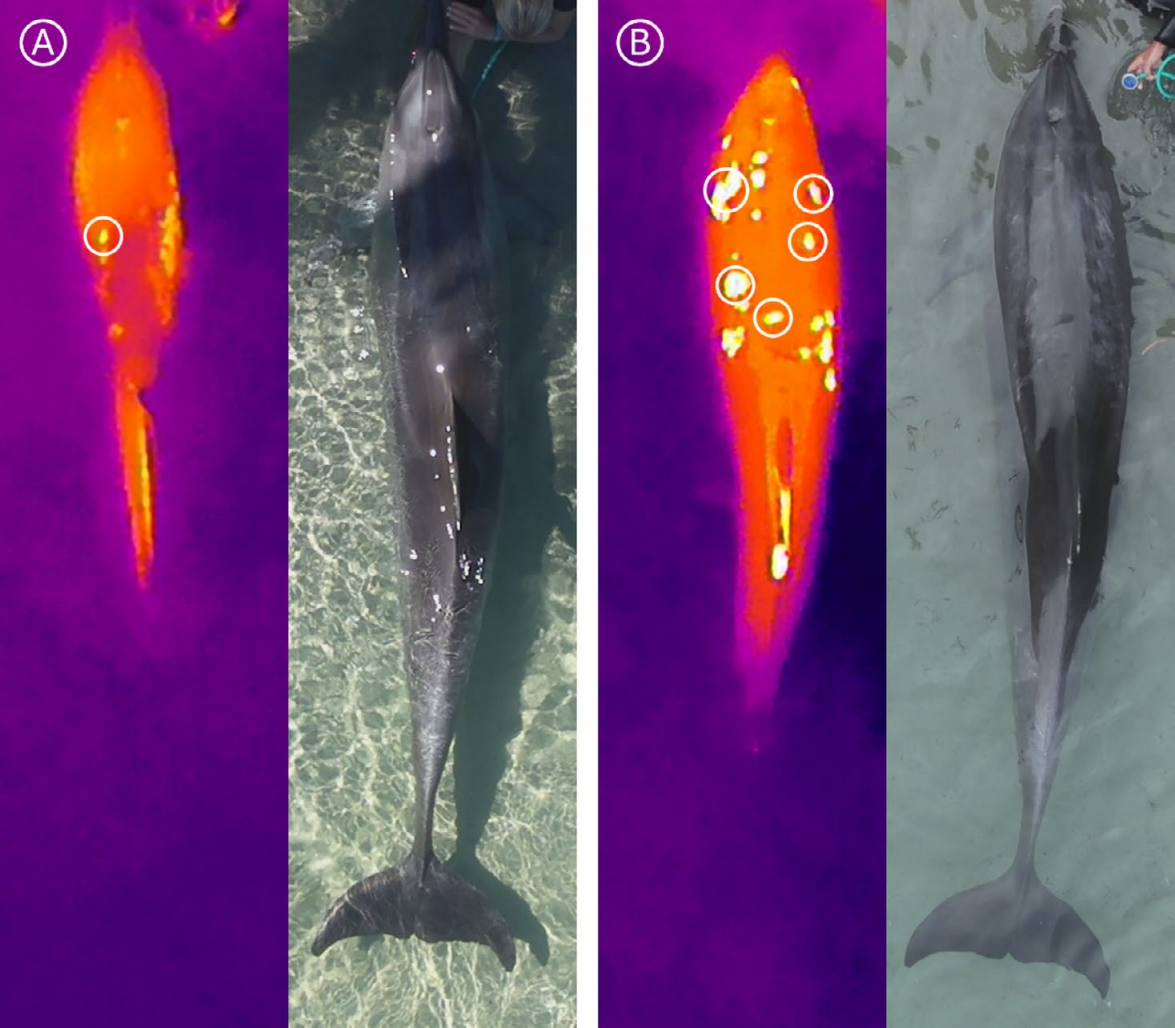
Infrared thermography (left) and paired RGB image (right) of an adult female bottlenose dolphin (TF5) recorded during summer (A) and winter 2023 (B) at Sea World, Gold Coast, Australia, captured from a flight height of 5 m and subsequently cropped and zoomed for visual clarity. In summer (A), thermal anomalies were present but relatively limited in extent, with a few discrete hotspots coinciding with external markings. By winter (B), anomalies were more numerous and pronounced, covering a larger proportion of the body surface and corresponding with extensive visible markings. Anomalies measured are shown in the white circles.

Drone‐IRT consistently identified localised hotspots that were significantly warmer than the surrounding body surface temperature in both dolphins. In TM5, a crescent‐shaped hotspot was visible behind his blowhole during the summer surveys, despite no obvious external markings at this time. By late summer, scratch marks appeared in the same region as the thermal anomaly. However, by winter, both the hotspot and associated markings had disappeared, suggesting the issue had resolved (Figure 1). In contrast, TF5 displayed continuous hotspots during summer, but these became more numerous and prominent in winter, covering a larger portion of the body surface and coinciding with more extensive external skin markings, potentially demonstrating an increase in skin irritation (Figure 2).

For TM5, hotspots averaged 3.6°C ± 0.27°C (x̄ ± SD) warmer than surrounding body surface temperatures. Hotspot temperatures ranged from 34.0°C to 35.7°C, whereas body surface temperatures remained tightly constrained between 30.9°C to 31.9°C. Statistical testing confirmed this difference to be highly significant (*p* < 0.001). For TF5, seasonal comparisons revealed consistent but variable hotspot anomalies. During summer, hotspots were on average 3.2°C ± 0.03°C warmer than the surrounding body surface (*p* < 0.001). Hotspot temperatures ranged from 32.9°C to 34.1°C, while body surface temperature values ranged from 29.8°C to 30.6°C. In winter, anomaly difference increased to 4.8°C ± 0.35°C, again highly significant (*p* < 0.001). Hotspot temperatures ranged from 29.6°C to 31.4°C, whereas body surface temperatures ranged from 25.2°C to 26.8°C. This seasonal contrast indicates that TF5's hotspots were persistent across both survey periods, but more pronounced during the winter survey period. Together these results demonstrate that drone‐IRT can detect consistent hotspot anomalies in dolphins, with the anomalies being clearly distinguishable from surrounding body temperatures in both individuals. Specifically, hotspots were 3°C–5°C warmer, indicating that drone‐IRT can reliably distinguish hotspot regions from baseline body surface temperatures.

These consistent patterns indicate that the anomalies were not random but repeatable, biologically meaningful features, suggesting underlying physiological processes such as transient irritation or tissue recovery rather than incidental variation. However, in the absence of clinical or diagnostic confirmation, these thermal anomalies may also reflect disruption of the insulating properties of the skin or normal variation in heat off‐loading associated with thermoregulation, processes that remain incompletely characterised in cetaceans (Favilla and Costa 2020; Noren et al. 1999). These results also highlight the potential for temporal and seasonal variation in hotspot patterns. In TF5, anomalies were consistently present across both summer and winter surveys, but the magnitude was greater during winter. Notably, the hotspots and associated body markings were also more visible in winter, suggesting that the contrast may have been enhanced as a result of tissue irritation than just environmental conditions. Cooler water and air temperatures lower baseline skin values, increasing the relative difference between inflamed tissue and surrounding areas, thereby making anomalies more conspicuous (Fischer Verlag et al. 1998; McCafferty et al. 2011). Alternatively, the seasonal progression of irritation may have contributed to changes in the absolute intensity of the hotspot anomaly (Mota‐Rojas et al. 2024). Although this dataset is limited to two dolphins, the consistency of the results suggests that such thermal signatures can provide biologically meaningful indicators for assessing tissue status and healing dynamics (Fridberg et al. 2024).

These observations complement previous applications of IRT in veterinary and wildlife contexts, where localised surface temperature changes have been limited to inflammation, infection or scarring (Fridberg et al. 2024; McCafferty 2007). For TM5, the hotspot coincided with scratch marks that later resolved, possibly consistent with transient inflammation in animals (McManus et al. 2016). For TF5, hotspot anomalies persisted across seasons and were associated with more extensive physical markings throughout winter, consistent with either ongoing irritation (Lonati et al. 2022) or altered thermoregulatory properties of scarred skin (Norris et al. 2010). Such associations highlight the potential for drone‐IRT to move beyond visual documentary of scratches and scarring to detect the underlying physiological processes associated with injury and recovery, though further in‐depth studies are required.

This study demonstrates that thermal imaging from drones can provide reliable measurements under controlled conditions. All hotspots were detected at 10 m altitude, and paired RGB imagery enabled confirmation of their alignment with external markings. Importantly, the approach was entirely non‐invasive and caused no detectable disturbance to the animals, emphasising its potential suitability for health monitoring in free‐ranging cetaceans in non‐controlled conditions, warranting further investigation. No behavioural abnormalities or husbandry differences were noted for TM5 or TF5, and both were maintained under identical environmental conditions to the other dolphins. Beyond drones, IRT also holds value as a stand‐alone tool, with potential applications for monitoring dolphins and other cetaceans within human care, where close‐range imaging could support veterinary assessments (McCafferty 2007; Melero et al. 2015; Russell et al. 2024).

Limitations remain, particularly the absence of veterinary ground‐truthing to confirm the physiological basis of the anomalies. Although drone‐IRT detected consistent hotspots, linking these anomalies to specific causes requires veterinary expertise and diagnostic confirmation. The small sample size also limits broader applicability, and further studies incorporating clinical validation and more individuals will be essential to establish the biological meaning of hotspot anomalies and their wider applicability.

Despite these limitations, the results demonstrate the feasibility and potential for drone‐IRT as a novel tool for monitoring cetacean health and skin condition. By revealing localised thermal anomalies not always apparent through visual surveys, this approach extends existing health monitoring techniques and aligns with the growing use of drones in marine mammal research.

## Author Contributions

**Charlie White:** conceptualization (equal), data curation (lead), formal analysis (lead), investigation (lead), methodology (equal), writing – original draft (lead), writing – review and editing (equal). **Andrew P. Colefax:** conceptualization (equal), data curation (supporting), methodology (equal), supervision (equal), writing – review and editing (equal). **Guido J. Parra:** conceptualization (equal), funding acquisition (lead), methodology (equal), supervision (equal), writing – review and editing (equal).

## Funding

This work was funded by the Cetacean Ecology, Behaviour and Evolution Lab, College of Science and Engineering, Flinders University, Adelaide, South Australia.

## Conflicts of Interest

The authors declare no conflicts of interest.

## Data Availability

Data and code used for this study are available from Figshare: https://doi.org/10.6084/m9.figshare.30977104.
